# The value of uncertainty in critical illness? An ethnographic study of patterns and conflicts in care and decision-making trajectories

**DOI:** 10.1186/s12871-016-0177-2

**Published:** 2016-02-09

**Authors:** I. J. Higginson, C. Rumble, C. Shipman, J. Koffman, K. E. Sleeman, M. Morgan, P. Hopkins, J. Noble, W. Bernal, S. Leonard, O. Dampier, W. Prentice, R. Burman, M. Costantini

**Affiliations:** 1 King’s College London, Cicely Saunders Institute, Department of Palliative Care, Policy and Rehabilitation, Bessemer Road, Denmark Hill, London, SE5 9PJ UK; 2 King’s College London, Department of Primary Care and Public Health Sciences, Capital House, London Bridge, London, UK; 3 King’s College Hospital, King’s Critical Care, Denmark Hill, London, UK; 4 Palliative Care Unit, IRCCS Arcispedale S. Maria Nuova, Reggio Emilia, Italy

**Keywords:** End-of-life care, Palliative care, Intensive care unit, Critical care, Decision-making, Uncertainty, Pathways, DNACPR

## Abstract

**Background:**

With increasingly intensive treatments and population ageing, more people face complex treatment and care decisions. We explored patterns of the decision-making processes during critical care, and sources of conflict and resolution.

**Methods:**

Ethnographic study in two Intensive Care Units (ICUs) in an inner city hospital comprising: non-participant observation of general care and decisions, followed by case studies where treatment limitation decisions, comfort care and/or end of life discussions were occurring. These involved: semi-structured interviews with consenting families, where possible, patients; direct observations of care; and review of medical records.

**Results:**

Initial non-participant observation included daytime, evenings, nights and weekends. The cases were 16 patients with varied diagnoses, aged 19-87 years; 19 family members were interviewed, aged 30-73 years. Cases were observed for <1 to 156 days (median 22), depending on length of ICU admission. Decisions were made serially over the whole trajectory, usually several days or weeks. We identified four trajectories with distinct patterns: curative care from admission; oscillating curative and comfort care; shift to comfort care; comfort care from admission. Some families considered decision-making a negative concept and preferred uncertainty. Conflict occurred most commonly in the trajectories with oscillating curative and comfort care. Conflict also occurred inside clinical teams. Families were most often involved in decision-making regarding care outcomes and seemed to find it easier when patients switched definitively from curative to comfort care. We found eight categories of decision-making; three related to the care outcomes (aim, place, response to needs) and five to the care processes (resuscitation, decision support, medications/fluids, monitoring/interventions, other specialty involvement).

**Conclusions:**

Decision-making in critical illness involves a web of discussions regarding the potential outcomes and processes of care, across the whole disease trajectory. When measures oscillate between curative and comfort there is greatest conflict. This suggests a need to support early communication, especially around values and preferred care outcomes, from which other decisions follow, including DNAR. Offering further support, possibly with expert palliative care, communication, and discussion of ‘trial of treatment’ may be beneficial at this time, rather than waiting until the ‘end of life’.

## Background

With increasingly intensive medical treatments available and population ageing, caring for critically ill patients facing complex treatment and care decisions will become more common [1–3]. In response, intensive care bed provision is expanding in many countries [1, 2] affecting medical and surgical practice. Consequently, many deaths occur in intensive care units (ICUs), often preceded by a decision to limit or withhold life-sustaining treatment, [4] and palliative care support for this patient group has been proposed [5]. A multinational cohort study (SAPS 3) found an in-hospital mortality of 21 % for ICU patients [6] with 8.6 % of patients having a decision being made to forego life-sustaining treatment (including both withholding and withdrawing treatment) and 36 % of deaths occurring after such a decision. Other studies suggest a much higher figure of 60-80 % of ICU deaths occurring after limitation of treatment [7, 8].

Decision-making in this context is complex. Patients with critical illness are often unable to participate in decision-making because of the severity of illness and consequent lack of capacity [9]. Those close to the patient are therefore frequently informed and involved, to varying degrees, in the decision-making process on their behalf. Lautrette et al. [10] suggest that intense communication is required with those close to the patient and, where possible, the patient themselves, with the decision-making process tailor-made to individual preferences. Communication and skilful management of decision-making is often key to family and patient satisfaction [11, 12]. However, while much research has considered family members’ preferences for involvement in decision-making, ‘decision-making’ is considered often as a single entity. We do not know how the trajectory of illness, and the different decisions involved, affect preferences, or where conflicts arise. Therefore, we aimed to explore the nature and patterns of decision-making processes during the whole trajectory of ICU admissions, including sources of conflict and resolution.

## Methods

### Design

Prospective ethnographic study: a case study approach included non-participant general observation on ICU, followed by selection of cases, with qualitative interviews and direct observations. Ethical approval was granted by the South East London Research Ethics Committee (08/H0805/65) - the hospital Research and Development department gave governance approval and monitored the study conduct. We followed MORECARE research recommendations [13, 14]. We first undertook general observation on the ICUs (with no identification of individuals). This informed future methods.

For the case studies, we gained informed consent where possible from patients. For patients who lacked mental capacity, we followed the Mental Capacity Act (MCA) of England and Wales (2005). When approaching patients, researchers took the view that capacity for the decision to participate in research should be assumed to be present, unless shown to be absent. We took steps to check if there were times when patients may have had capacity and explored ways in which capacity might be improved and discussed this with clinical teams. However, we anticipated that many patients were likely to lack capacity (often being unconscious). In these instances, as recommended by the MCA, we identified and then approached a ‘personal consultee’ who knew the patient, was not acting in a professional or paid capacity, and could be trusted to advise on what the patient would have wished. This was usually a family member or close friend. The consultee gave advice on whether the patient would have wanted to take part, and we sought their assent for the patient to be involved in the study. Under the MCA, where consent would normally be obtained, no individual can consent on behalf of another about participation in a research study, unless it is a medicinal trial. Where no ‘personal consultee’ could be identified, we attempted to seek a ‘nominated consultee’ (for example a health care professional, usually the patient’s Consultant) to advise on a person’s likely views and interests. If their Consultant was a member of the Project team, we approached another appropriate Consultant, with no connection to the research study. Family members also gave informed consent for their own participation in interviews and any observation. Following consent/assent we observed care, communication and decision-making processes; reviewed clinical records; and interviewed the family member (and/or patient). Strict principles of confidentiality, anonymity and data protection were followed by all the study team.

### Setting

Two ICUs (one general, 33 beds, grouped 18 + 15; one specialist liver disease, 14 beds) in an inner-city hospital serving an ethnically and socially heterogeneous population [15]. The ICUs treated all categories of critical illness other than burns, including brain injury, cardiovascular, transplantation, vascular, general and specialised medicine. The ICU has an SMR of 0.85 in the ICNARC model [16] and is a closed unit with a governance process around protocolisation and guidelines. The hospital also has a multiprofessional specialist palliative care team, which works closely with ICU. Palliative Medicine has been a specialty in the UK since 1987, and has recently introduced an exit examination.

### Subjects

Over six months we selected a purposive sample of patient cases to achieve maximum variation in patient characteristics (ethnic group, age, gender etc.) and thus in perceptions and experiences [17]. We included patients where doctors or nurses on ICU identified there were potential end of life concerns, discussions or a high risk of dying during the current admission. The ICU consultant in charge of the patient’s care, together with the lead nurse, assessed both the capacity of the patient to consent and their ability to participate. If the patient was deemed unable to consent, assent was gained from a family representative for observation and review of medical records. Family members were identified via patients and clinicians and gave individual informed consent for their interviews.

### Data collection

In the non-participant general observation of ICU activity two researchers were based on the units, usually in the nurses’ station or reception area, at different times of the day/night. They collected information on the layout of the units, activity levels, flow of admissions and visitors, clinicians attending, ward rounds, team meetings and family conferences, and the types of decisions occurring. No individual patients or families were identified in this phase.

For the case studies we collected three sources of data:

1. Face-to-face semi-structured interviews with a family member or friend close to the patient in a quiet room at a time convenient to the participant. Interviews were based on a topic guide (including thoughts about communication, interactions, decision-making, and perceptions of care), recorded and transcribed verbatim.
2. Non-participant observation. Two researchers observed care and attended ward rounds, case discussions and team meetings. They collected data using standard recording sheets, which included information on the layout of the unit affecting the patient, activity levels, patient condition, visitors, patient care, professional activity and interactions. Observations were carried out at different times of day, on different days of the week, when different staff were on duty and continued until the patient’s death, discharge or recovery.
3. We reviewed medical records and extracted data onto a standard recording sheet including, documentation relating to decision-making and end of life issues.

All data were anonymised. Code numbers were allocated to each case. Validity and reliability was enhanced by development of topic guides and observation logs with an independent service user group, and piloting in ICU.

### Analysis

Transcripts were double-coded by CS and CR and analysed using content analysis. A re-iterant process of discussing areas of agreement and disagreement took place to achieve consensus. Data from the initial non-participant observation were integrated with the case study findings. Alternative interpretations were incorporated. The analysis was further tested during discussions with colleagues and meetings with other members of the research and ICU teams.

We explored all decisions made during ICU admissions to identify the trajectory of decision-making, and attempted to pinpoint two key decisions identified as potentially important in earlier literature:

- That a patient was at high risk of dying, which might act as a trigger to using an end of life care intervention (D1);
- The decision to withdraw any life sustaining treatment (D2).

We compared all decisions between cases and within themes. For each case, every decision that could be identified was tabulated and coded into emerging sub-types of decision-making. We identified the general aim of care with each of these individual decisions and coded them as decisions made with curative intent (or in line with clinical improvement/prolonging life) or decisions made for comfort care (including for quality of life or symptom control). We then identified preferences for involvement and any disagreement or conflict apparent between any of those involved in the decision-making process (patient, those close to the patient, ICU staff, or referring clinicians / other teams). Framework tables were compared between cases and within cases over time to identify patterns in decision-making. We provide three illustrative case examples to portray the nature of trajectories. Details were removed or amalgamated from different cases to preserve anonymity.

## Results

The initial non-participant observation included all times of day, including, evenings, nights and weekends. For the cases, we included 16 patients with 19 family members or relatives (Table 1); 3 patients had two family members. Of these, 6 patients were alive six months after the end of the study period; ten had died. Twelve had an identifiable D1 and four a D2. The remaining four of the 16 patients had been deemed suitable for inclusion in the study so therefore had been identified as having end of life issues or discussions taking place, although D1 could not be clearly pinpointed. We conducted 249 h of non-participant observation: 44 in the initial general phase; 205 of care relating to the specific cases.

**Table 1:** Demographic characteristics of the 16 patient cases involved in the study, for which 19 family members were interviewed

Age of patients (median, range)	50 (19 – 87) years
Gender of patients	8 women; 8 men
Diagnosis	3 Infective
	1 Hypoxic brain injury
	4 Neurological injury
	3 Malignancy
	2 Gastrointestinal
	2 Organ Transplant
	1 Organ failure
Total length of ICU stay	22 (1-156) days
median (range)	Three patients had two admissions
Gender of family members who provided information	10 women; 9 men
Age of interviewees (range)	53 (30 – 73) years
Ethnicity of interviewee	16 White British
	2 Mixed Caribbean
	1 Afro-Caribbean
Religion of interviewee	9 No religion
	8 Christian
	1 Muslim
	1 Other
Relationship to patient	8 partner/spouse
	7 son/daughter
	4 parent

### Trajectory of decision-making during the ICU admission

We identified four main trajectories of decision making for patients in the ICU, with distinct processes and conflicts. There was no observable pattern in terms of age, diagnosis, ethnicity or religion across the groups.

#### Aim of care remained curative / life prolonging throughout the admission

Seven cases were in this group, including all four cases where a clear D1 could not be identified. For these four patients, all decisions made were for active management and they all survived to be discharged from ICU, although two died within three months of discharge. Three further cases in this subset had the high risk of death explained to the family (D1). There was a wide variation of length of stay on the unit from 6 to 156 days. No limits to treatment were set in any of these cases. There were active decisions throughout the admission and care was described as supportive (with multiple organs being supported – all had renal replacement therapy, cardiovascular support and were ventilated).

### Preferences and involvement in decision-making

Of the three cases where D1 could be assigned, the patients did not have capacity at this time. They all improved clinically, regained capacity later in the admission and were discharged to the ward. Many decisions were made day-to-day in each of these cases and generally there was not clear documentation of who was involved in the decision-making process, although from general observations, decisions appeared to be directed by the medical team and not shared with the relatives. The exception to this was the decision for transplantation: in the two cases where this was required there was a documented shared decision-making process with the family. The relative interviewed in one case described his participation in decision-making for active treatment. The relative suggested that since the patient was physically unable to sign the form, he felt he had no option but to sign himself:

*He (consultant) said this is the only way forward...... I said “He can’t even lift a pen up” … he said “well somebody’s got to sign for it”, “I’ll sign for it” I said, because I want to see him go forward and, he said “right, okay, well we’ll fill the forms in” and I signed it.* (Interview transcript)

In the other case the staff seemed, initially, to require the family to ask the right question to initiate their involvement in decision-making:

*We didn’t discuss* [surgery] *as family didn’t ask.* (Medical Record)

Two days later the clinicians again spoke with the family and subsequently the patient was re-listed for transplant with full involvement in this decision.

In this group there was little evidence of conflict documented, observed or discussed by the interviewees.

#### Shift from curative to comfort care

Three cases were in this group, all had an identifiable D1 and D2 and all died (see below for an illustrative example). The timings of decisions made in these three cases varied: two had a D1 at or soon after admission (day 0 or 1), whilst the third happened around day twelve of the ICU admission. All required respiratory support and had a tracheostomy performed and all received inotropes; one had renal replacement therapy. Two of the three subsequently had both limits to respiratory support and decisions made to withhold renal replacement therapy if required (one also had a treatment limit made that they would not be given inotropes if required). Two had a clear withdrawal of active treatment (D2) 3 days before death, whilst in the third case the patient survived for 48 days after a decision to withdraw active treatment (in this case organ support, though nutrition and fluids plus all comfort measures continued). All three cases had a documented aim of care as being comfort/symptom control at time of D2. Two cases had a formal Do Not Attempt (Cardio Pulmonary) Resuscitation (DNA(CP)R) decision documented (at or soon after admission), and both were referred to palliative care services.

### Example case of shift to comfort care (illustrative)

This lady was admitted to the unit in respiratory failure secondary to pneumonia. A decision was made by the intensive care consultant the day after admission to perform a tracheostomy. This decision was qualified with documentation that it would not preclude a reversal of the decision to provide respiratory support if it became evident that the respiratory failure was irreversible or if multisystem organ failure ensued. On this day a DNA(CP)R order was signed but full active treatment was continued, including cardiovascular support with inotropes and respiratory support. Documentation of discussions with the family varied with some very detailed entries and others more limited regarding content of conversations, people present, and whether there was any disagreement.

She had fluctuating mental capacity and at times was able to be involved in discussions and decision-making and she expressed that she would rather be pain-free and sedated. Difficulty in ventilating and an inability to wean respiratory support seemed to precipitate the shift from active to comfort care. Medications were streamlined in the last day of life to those for symptom control and blood tests were ceased, although monitoring continued. She died in a side-room with her family present 25 days after ICU admission.

### Preferences and involvement

In two of these cases there was opportunity for the patient to be involved in decision-making and both had an active role – one opting for surgical treatment and the other requesting symptoms to be controlled at the expense of conscious level. In this group there was a general sense of trust by the families in the staff making decisions, and belief that they will do the best they can according to their medical knowledge. Previous experience influenced preferences for one family, affecting their views regarding place of care. There was a sense that “decision-making” was considered a negative process by those close to the patient. When asked about their involvement in decision-making, these family members responded with words like “*dreading*” and *“touch wood she hasn’t really had to have any big decisions”.* Predicted or actual distress influenced staff in how much and at what stage they involved the patient or families in decision-making. For example, an ICU consultant expressed concern that discussing a decision to withhold organ replacement therapy might cause unnecessary distress, so it was not discussed with the relatives. The level of involvement was increased by direct prompts to staff such as expressions of views by patients (to optimise symptom control) and relatives (place of care).

#### Oscillating curative and comfort care in the admission

Five patients were in this group; characterised by a fluctuating illness trajectory and fluctuating capacity. All cases had an identifiable D1, and all had DNA(CP)R decisions documented. In one case a decision was made not for prolonged resuscitation and all had other limitations to treatment but with continuing active management.

Quality of life issues were identified and discussed in this group more than the others. For example, a decision was made to allow one patient to eat and drink accepting the respiratory risk associated with this; for another a relative questioned continuing curative management considering the potential reduction in quality of life; for a third patient a description of recent good quality of life was the factor that led to the reversal of a decision not to intubate and ventilate.

In all cases there was a conflict or disagreement and often a process of negotiation in at least one decision-making process. Different views were held by the ICU staff caring for the patient, and, either another member of staff, the patient or relative. Views and wishes also changed during the admission. One patient in this group had previously expressed to his family that he would not want to be resuscitated and they relayed this to staff, leading to the completion of a DNA(CP)R order. Later in the admission, staff and relatives agreed not to escalate treatment, but when the patient was subsequently able to talk (on day 12) he said he wanted full aggressive treatment. Limits to treatment were removed and he was documented as being for full treatment on Day 14. However, his condition deteriorated once more with a lowered conscious level on Day 16 and limits were again set and it was decided (with agreement of his family) that he was not for escalation of treatment. In another case, the family requested curative treatment be continued but the ICU consultant felt that this would not be beneficial, and the aim of treatment should become palliative. In a third case, the referring team felt that respiratory support should not be escalated above Non-Invasive-Ventilation (NIV), but when the patient was asked about his wishes he responded that he wanted full active treatment including cardio-pulmonary resuscitation and tracheostomy insertion, and these wishes were followed. The patient was discharged but subsequently re-admitted with an infection and ventilated, but limits were set again as not for multi-organ support or for prolonged resuscitation.

In the fourth case a decision was made not to intubate or ventilate. However, later on the same day this decision was reversed when another clinician expressed that they felt a trial of intubation and ventilation was indicated. In the fifth case, there was conflict between the ICU consultant and the relatives. An ICU consultant who had not previously been responsible for the patient’s care assumed a direct role in decision-making and signed a DNA(CP)R order at the time of discharge from ICU. In this case, the relatives were not satisfied with this decision as it had not been made at the multidisciplinary team meeting where other decisions were made. After negotiation it was agreed that the decision would be reviewed in two weeks. In this case, the relatives described different information coming from different staff:

*…. had his trache reduced about four or five weeks ago and we were told that’s a progression to taking that out, and we understand why it’s not come out, because he’s still got the chest infections, etc. But then on Wednesday, we were told by [ICU consultant] that he would never have his trache out and that came like a ‘where’s that one come from?’ And there were one or two other small things in that conversation and we were saying “well, that’s not what [neurosurgical consultant] is saying”* [Interview transcript]

DNA(CP)R orders, especially in those cases where patients did not have capacity, tended to be decisions made by medical staff with the relatives being informed e.g. the ICU consultant documents in one case “*He* [the relative] *knows I have written a DNAR order”* and two days later documents that he has not “*had the time to talk to the patient herself about progress and what she wants”.*

### Examples of illustrative cases of oscillating curative and comfort care

#### Case 1

This gentleman was admitted following a fall and sustaining a spine injury. He underwent surgery but remained quadriplegic and ventilator dependent. On day 2 of this admission the neurosurgical team decided not to escalate above non-invasive ventilation with the agreement of the ICU consultant. He had capacity to be involved with decision-making about his care at this time and his condition was discussed with him. He was asked if he wanted to continue having ventilatory support, in which case they would do a tracheostomy. He expressed that he wanted full active treatment, including resuscitation, and a tracheostomy was subsequently performed. He was discharged from ICU but re-admitted in respiratory failure secondary to infection. On the first day of this second admission it was documented that his resuscitation status was to be discussed with his family and the neurosurgeons and that prolonged resuscitation or multi-organ support was inappropriate. The next day the ICU consultant discussed the situation with the family and emphasised that, although he had improved slightly, he still had a serious spinal injury and the chance of any useful function return was very small and that he would not live as long as he would have without the injury. His condition continued to fluctuate; he was eventually discharged to a rehabilitation unit.

#### Case 2

This lady was admitted with an infective exacerbation of chronic obstructive pulmonary disease. On admission, the ICU consultant made the decision that she was not for intubation or resuscitation because of her poor prognosis and pre-admission condition. This was discussed with her family but they were still keen for her to be treated actively. She was then reviewed by a respiratory consultant who felt that, as there was some evidence of reversibility and because of her previous reasonable exercise tolerance and quality of life, she deserved a trial of intubation and ventilation, if required physiologically. The ICU consultant then documented that after the discussion with the family, the decision was made to proceed with intubation and invasive ventilation, but with the proviso to limit intensive care interventions to treat breathing failure only, and not to add in treatment for failure of other organs should this occur. He also mentioned possible scenarios of continuing deterioration and of weaning failure in which case he would stop ineffective treatment and allow her to die. On Day 3, a respiratory physician who had known the patient for some time reviewed her and agreed that treating her respiratory disease was in the patient’s best interests. This patient gradually improved and was discharged from ICU 24 days after admission.

### Preferences and involvement

Alongside capacity to be involved, communication barriers also had an impact; the level of involvement of one of the patients in this group was increased when the patient was able to speak.

Similar to Group A, the theme that families did not want to make medical decisions because they did not have sufficient knowledge emerged. The role that the family felt they needed to fulfil also affected how they felt they should be involved in decision-making. In one case the family member described how he needed to be a conduit of information to all other family members. This affected his involvement as he needed clear information to pass to others. Additionally in this case, previous experience of decision-making influenced preferences for involvement. Other relatives perceived disagreement and confusion about decisions being made earlier on in the admission and so opted not to be involved in a decision-making conversation.

For one of the cases in this group, religion was an important influence. The patient’s beliefs required him to express which treatments he would accept; these decisions were supported and emphasised by his family when he did not have capacity.

#### From admission onwards the aim of treatment is not curative but comfort

There was only one case in this group - an individual who died on ICU within 24 h of admission post-surgery. The ICU consultant documented that there was devastating brain damage and made the decision to discuss the case with the organ transplant co-ordinator. The decision was made not to attempt resuscitation but not to withdraw other support until there was a clear decision made by the neurosurgical team. Later in the afternoon the situation was discussed with his relatives and a consensus was reached for full withdrawal of life-prolonging therapies with a focus on end of life care. At this time all unnecessary medications and interventions were ceased if not required for donation. This patient did not have capacity at any point in the short ICU admission. A DNA(CP)R order was signed by the ICU consultant, and the relatives were involved in all other decisions. With respect to organ donation the staff fulfilled an informative role, whereas in other decisions (such as removal of the nasogastric tube and to cease unnecessary medications) they were more collaborative.

### Categories of decision-making

We identified eight categories of decision-making in our data which occurred throughout the disease trajectories. Five of these related to the process of care:

- Decisions about resuscitation status (e.g DNA(CP)R, or not for extended attempts of Cardio Pulmonary Resuscitation (CPR))
- Decisions around system support (e.g. invasive respiratory support, inotropes and/or vasopressor medication)
- Decisions around medications and fluids (e.g. antibiotics, analgesics, diuretics, neuroleptics)
- Decisions about interventions and monitoring (e.g. tracheostomy performed; no further blood tests)
- Decisions to involve other specialties / hold a multidisciplinary team meeting (e.g. refer to tissue viability nurse, surgeon, or palliative care; hold multidisciplinary meeting).

The other three categories of decision-making related to the outcomes of care:

- Decisions about aim of care (e.g. suitability for rehabilitation; whether to treat aggressively; focus management on palliation)
- Decisions about place of care (e.g. return to ward; discharge to ventilated bed rather than weaning unit)
- Decisions about care needs (e.g. not to be log rolled; needs attention to mouth care; needs support for family; needs pain monitoring and control).

Some decisions were coded in more than one category. For example, a decision relating to inotropes was coded as both a decision about system support and medication. Often these decisions in rapid succession, with, for example, decisions about aim of care, quickly followed by decisions regarding medications, DNA(CP)R etc.

Family members, and patients when they had capacity, were most often involved in decisions about outcomes and some treatments: i.e. place of care; specific interventions (especially if consent required; tracheostomy); withdrawing more aggressive treatment; rationalising of medications; medication for symptom management; aims of care; and holding a multidisciplinary team meeting. Others were more clearly directed by the clinician, e.g. starting organ support, active treatment decisions, future limitations to treatment, and other specialty involvement. These often followed discussions with patients and family members regarding care aims and needs.

## Discussion

In this study we found that decisions for critically ill patients were made serially throughout the ICU stay, usually over days and weeks. We did not identify a single linear trajectory. Instead we found four trajectories with different patterns of clinical decision-making: curative care from admission; oscillating curative and comfort care; shift to comfort care; and comfort care from admission. In two of these trajectories – curative care from admission and comfort care from admission – the decision-making had less conflict. When the condition of patients fluctuated and curative and comfort measures oscillated, we observed more conflict. Conflict occurred between relatives and staff and between and within clinical teams. Some patients and families considered decision-making as a negative concept, heralding deterioration. Relatives seemed to find it easier when patients switched definitively from curative to comfort care than when patients fluctuated. Decisions made covered many different aspects of care. We identified eight categories of decision-making; three related to the outcome of care (aim, place, response to needs) and five to the process of care (resuscitation, decision support, medications/fluids, monitoring/interventions, other specialties).

Our finding of distinct types of decisions associated with varying involvement runs contrary to most literature on patient or surrogate preferences for decision-making. Heyland et al found that most family members prefer some form of shared decision-making with physicians, but that such involvement is missing often for older patients leading to lower satisfaction [18]. Other research has considered the model of passive, shared and active roles, assumed by relatives of patients that do not have capacity [9, 19, 20]. All these suggest it is important for family members to identify which of these roles they should assume for the needs of each individual relative, including the culture and situation [21]. However, it is not clear which decisions are involved. Our study found that the preferred and actual involvement varies for different decisions. Families assumed differing roles, and were most involved in decisions related to care outcomes and aims. Family preferences for involvement varied but generally relatives wanted information and understanding of the process but not greater involvement in these (and less involvement was preferred by some). This finding also brings into question the current emphasis in many countries on a single specific decision of a Do Not Attempt (Cardiopulmonary) Resuscitation order (DNA(CP)R). In North America and much of Europe there is great emphasis on physicians discussing and documenting this specific decision [22]. However, this approach has recently been called into question, because of the negative frame of DNA(CP)R and the omission of goals of care, proposing instead an "Allow Natural Death" order [23]. Our data indicate: (a) excellent communication skills are needed, (b) individual preferences must be elicited and (c) discussions and involvement regarding decisions about the overall goals and outcomes of care are preferred over process decisions. Our data reinforce the step-wise model proposed by Limehouse et al, developed in the Emergency Department, which, once capacity is established, recommends: eliciting patient (and family) values; determining patient/surrogate understanding of the life-limiting event and expectant treatment goals; conveying physician understanding of the event, including prognosis; treatment options; followed by discussion and decisions regarding treatment goals including withdrawing or withholding resuscitative efforts [24].

An important finding from our analysis is that 6 out of the 16 patients in our study who were identified as having a high risk of dying did not die within six months of the end of the study period. This is in keeping with an analysis of 14,488 patients in 282 ICUs, which found that among those with a decision to forgo life-sustaining treatment, up to 20 % survived [6] and suggests our qualitative sample included a range of patients with regard to treatment outcomes. Patients are admitted to ICU because of profound and complex illness(es), and there is often uncertainty about prognosis indicating the need for both curative and comfort measures. Our findings support the proposals of Mosenthal et al and others, for integration of palliative care into critical care, [25] involving patients and relatives early in decisions where possible [5, 26]. They also bolster the work of Levin et al, which recommends skilled communication and family meetings [27]. Our results emphasise the value of approaches that use tools to aid understanding of patient and family views and wishes on admission to ICU, as in the Psychosocial Assessment and Communication Evaluation (PACE), [15] rather than the adoption of predesignated care pathways [28]. It is of note that some families regarded decision-making as a negative concept, heralding impending deterioration, and instead preferred uncertainty. A recent trial by Bruera and colleagues suggests that doctors who impart ‘bad news’ are judged less empathetic than those with ‘good news’ even when their behaviour is exactly the same [29]. Thus, periods of uncertainty and any shift to ‘bad news’ may be especially challenging, requiring additional skills and excellent communication from the clinical team [30].

Although critically ill patients frequently do not have capacity, this is often fluctuating. We found some patients had periods of lucidity when it was appropriate to involve them in the decision-making process. Lautrette et al suggest that intense communication is required with those close to the patient and the patient themselves, where possible, with decision-making processes tailored to their particular preferences [10]. Truog proposes that the focus in the USA is for the patient to identify a person who can act as surrogate decision-maker, whereas in Europe, physicians often make decisions when patients do not have capacity, in consultation with the family [31].

The longitudinal nature of our data collection allowed decision-making processes to be examined over time. In the ICU there is a high risk of mortality by definition, and this is often explained to the patient and/or relatives. Therefore all patients could potentially be at risk of dying (D1). However, admission to the ICU is an active decision for potentially curative treatment. A period of assessment often occurred during the early stages of the ICU admission when curative management continued, described by clinicians as a time of waiting for the patient to “declare themselves”, with organ support provided until it was clearer how the patient would respond. There was then either an increase in the role of symptom management with a varying decrease in curative management or else continuation of curative management. The aims of care however were not always clear as, on occasion, system support was continued for symptom control purposes and additionally some decisions were made that could be considered as both curative and for symptom control, e.g. a tracheostomy being performed.

This dual aim of care and cure may occur frequently in the ICU and some other settings because of the uncertainty involved in prognosticating and hoping for the best whilst preparing for the worst. It was in cases with an oscillating trajectory that most conflict arose, therefore, where palliative care specialists with their expertise in communication, symptom control, holistic approaches, support for families and care planning may be most valuable [32–35]. Their role may be in supporting or working with existing teams, to reach agreement, as well as working with patients and families. However, the exact model of ideal involvement is not yet clear. Our data support involving palliative care teams early, particularly during oscillating trajectories, may be particularly helpful, rather than waiting until the very end of life. This is consistent with evidence in other settings that early involvement of palliative care is valuable in improving satisfaction, quality of life, and decision-making [33, 36]. In ICU settings, where staff have expert communication skills, the best model of support, whether direct or indirect, requires further evaluation. We have developed a tool, PACE, to help all ICU clinicians provide good psychosocial assessments and communication [15]. Such a tool may help staff to support families and patients during clinical uncertainty and when patients deteriorate [37]. Other approaches have included more consultative or integrative models [38]. Our data suggest that the involvement of palliative care should not be all or nothing in ICU, it is vital to build up relationships between the services and to improve integration [39] to enable more sophisticated approaches matched to patient and family need.

It is also likely that some patients (mainly group C) warrant a trial of treatment and reassessment of goals of care; this trend is already occurring in many ICUs. The ICUs in the study analyse and record every death using a modified version of the ETHICUS definitions [4]. Through this they have identified changes in practice and decision-making over the last 10 years. There is an increasing tendency to use a 'trial of treatment' strategy even in patients with significant pre-existing disability and comorbidities. The team are now making far fewer treatment limitation decisions based on subjective criteria such as age, chronic disease, and quality of life.

The focus of our study was the nature and patterns in decision-making processes, and sources of conflict and resolution. The study was not intended nor designed to audit specific clinical actions and our findings should not be interpreted as such. For example, we report a case where a tracheostomy was inserted on day 1. There is no consensus on the optimal timing for a tracheostomy; randomised trials and systematic reviews comparing early versus later timing have shown no clear difference, and the practice of staff in this study was to follow current evidence-based recommendations for best practice [40, 41]. These are that a decision to place a tracheostomy should be individualised, depending on the clinical context and best interests of a patient and not on an arbitrary time in ICU [40]. There may be patients where it is clear they will need a tracheostomy on day 1. However, there are many other patients where a more detailed consideration is required; which can take several days or weeks. Equally, we noted an instance when surgical options were not initially discussed with the family as "they didn't raise it"; they were discussed two days later. This delay in discussion could be interpreted as demonstrating inadequate consent or shared decision-making. It is known that many doctors and nurses find initiating such discussions difficult in ICU [42]. However, fuller individual circumstances would be needed to judge the ‘quality’ of this particular instance; and these are not available for ethical and confidentiality reasons.

At a local level, our research collaboration between ICU and palliative care, and the study findings, has provided impetus for local improvements to service provision. The ICU has been expanded to 76 beds merged in one unit, with a similar expansion in staffing. The issues identified in this study have prompted plans for a new clinical information system, with improvements to handover and inter-professional communication, supported by an improvement science collaboration. Our study findings have also informed the design of a new build ICU. Clinically, a full time palliative care social worker is now based in ICU, to provide additional help and support during some of the difficult communication, decision-making and psychosocial issues.

A limitation of this study is that the data collected does not capture every aspect of the decision-making process as observations were not performed 24 h a day. However, we aimed to reduce this potential bias by collecting data from different sources, the medical record, the relatives, the staff, and by direct observation. However, there will be some situations where discussions occurred but were not documented in the medical record or directly observed. We carried out non-participation observation which involved the two researchers observing activity whilst not becoming part of the formal organization of the unit. Staff members were aware of the presence of the researchers, and at times we felt that this influenced their behaviours. For example, they sometimes asked the researchers if they thought a particular patient should be referred to palliative care. However, over time the researchers became accepted as a part of normal life on the unit and thus had less impact.

A further limitation is that the study was conducted in two ICU’s in a single hospital. It may be that decision-making processes and wishes are different elsewhere. However, our strengths were that the area served is culturally diverse, as represented in our sample, and that we included a broad mix of ages, diagnoses and length of ICU stay. However, only 3/19 interviewees were of Mixed Caribbean or African Caribbean ethnicity. Decision-making and information sharing practices vary between and within ethnic and cultural groups, [43] and further work is needed to understand if our results apply to a broader range of cultures. It would be useful also to have more information about income and education, especially since the median length of ICU stay was long and some individuals may have been financially unable to remain at their relative’s bedside to be involved in decision-making. Inclusion of some patients who oscillated between ICU and general wards may suggest that our findings apply beyond ICU. With the ageing population, [44] understanding decision-making for people with complex comorbidities, whether on or off the ICU, will become increasingly relevant.

Finally, there was only one patient in group D identified as receiving comfort care from time of admission. It may be that this case was unique rather than representing a sub-group of patients. We cannot be sure that the lack of conflict found was truly reflective of this situation or whether other families might hope for a miracle and then express anger at the treatment team. A challenge is that patients in this group are likely to be in ICU for only a short time and therefore are more challenging to include in research where consent processes often take time. Retrospective survey or case note review might be a way to assess this further.

## Conclusions

There is a complex web in the trajectory of decision-making for critically ill patients. Decisions for people in ICU are made across many different aspects of care, not just those relating to withholding or withdrawing life sustaining treatment. Our data suggest that emphasis should be placed on understanding patient and family member values and their involvement in decisions about the outcomes and goals of care, from which decisions about specific treatments and processes can flow. Patients and relatives had different preferences for involvement between individual cases and different types of decisions. The most complex and conflicted decisions were in cases of fluctuation and uncertainty, and in these instances our data suggest that high levels of consistent communication, understanding of individual preferences, flexibility, and in some cases, palliative care, are needed.
